# Elucidating *Spirocerca lupi* spread in the Americas by using phylogenetic and phylogeographic analyses

**DOI:** 10.3389/fpara.2023.1249593

**Published:** 2023-09-27

**Authors:** Paula Alfaro-Segura, Joby Robleto-Quesada, Víctor M. Montenegro-Hidalgo, Jose Arturo Molina-Mora, Gad Baneth, Guilherme G. Verocai, Roger I. Rodriguez-Vivas, Alicia Rojas

**Affiliations:** ^1^ Laboratory of Helminthology, Faculty of Microbiology, University of Costa Rica, San José, Costa Rica; ^2^ School of Veterinary Medicine, Universidad Nacional de Costa Rica, Heredia, Costa Rica; ^3^ Centro de Investigación en Enfermedades Tropicales, University of Costa Rica, San José, Costa Rica; ^4^ Koret School of Veterinary Medicine, The Hebrew University of Jerusalem, Rehovot, Israel; ^5^ Department of Veterinary Pathobiology, School of Veterinary Medicine & Biomedical Sciences, Texas A&M University, College Station, TX, United States; ^6^ Campus de Ciencias Biológicas y Agropecuarias, Facultad de Medicina Veterinaria y Zootecnia, Universidad Autónoma de Yucatán, Yucatán, Mexico

**Keywords:** *Spirocerca lupi*, phylogenetics, phylogeography, *Spirocerca vulpis*, migration

## Abstract

*Spirocerca lupi* is a parasitic nematode of domestic and wild canids of the world. This nematode induces esophageal spirocercosis and may eventually lead to carcinomas, aortic aneurisms, and death of the animal. Two genotypes of *S. lupi* have been described based on specimens from Europe, Asia, Africa, and Oceania, but no profound analysis has been conducted with *S. lupi* from the Americas. To study this, *S. lupi* specimens isolated from domestic dogs from Mexico, Costa Rica, and the United States, were molecularly characterized using 18S rDNA and *cox*1 fragments. Bayesian inference (BI) phylogenetic trees, Templeton-Crandall-Sing (TCS) haplotype networks and Principal coordinate analysis on nucleotide distances were constructed for each locus separately. In addition, a phylogeographic study using a fragment of the *cox*1 gene was used to infer the evolutionary history of the genus. BI *cox*1 trees grouped *S. lupi* from the Americas in genotype 1, together with Israeli specimens, and showed a high nucleotide identity with those worms. In the TCS network, American specimens clustered next to Israeli *S. lupi*. Furthermore, the 18S rDNA gene fragment separated Costa Rican worms from African, Asian, and European specimens and other species of the family Spiruridae. Interestingly, the phylogeographic analysis suggested that the origin of *S. vulpis* was in Europe, and it later diverged into *S. lupi* that spread first to Africa, then to Asia and finally to the Americas. Therefore, we suggest that the worms from the American continent might have originated from Asia by dispersion of infected intermediate, paratenic or definitive hosts.

## 1 Introduction

*Spirocerca lupi* is a parasitic nematode of mainly wild and domestic canids belonging to the order Spirurida, family Spirocercidae (Nascimento Gomes et al., 2021). This parasite has an indirect life cycle with coprophagous beetles as intermediate hosts, canids as definitive hosts, and other animals such as rats, lizards, or birds that can act as paratenic hosts (Rojas et al., 2020b). Canids become infected by eating beetles or paratenic hosts with the infective L3 larvae, which then migrate through the stomach, penetrate the gut wall, and travel through the gastric artery walls until they reach the aorta and the esophagus (Rojas et al., 2020b). In this way, *S. lupi* induces a disease known as spirocercosis, which usually presents as esophageal nodules that can progress from fibrous and inflammatory lesions to neoplastic lesions in the esophagus (Rojas et al., 2020b).

This parasite is distributed in the tropics and subtropics of the world, and extensive reports from Israel (Aroch et al., 2015), Southeast Asia (Aroch et al., 2015; Hoa et al., 2021), Africa (Greeff et al., 2018), and different regions of Europe (Giannelli et al., 2014; Psáder et al., 2017) have been published. In the Americas, *S. lupi* has been detected in Southern United States of America (USA) in coyotes (*Canis latrans)*, gray foxes (*Urocyon cinereoargenteus*), red foxes (*Vulpes vulpes*) and bobcats (*Lynx rufus*) (Pence and Stone, 1978; Morrison and Gier, 1979), in domestic dogs from USA (Pence and Stone, 1978), Mexico (Rodríguez-Vivas et al., 2019), Costa Rica (De Aguiar et al., 2021), and Brazil (Costa Santos et al., 2012), in maned wolves (*Chrysocyon brachyurus*) from Brazil (Blume et al., 2014), Andean foxes (*Lycalopex culpaeus*) from Peru (Gomez-Puerta et al., 2018) and Chile (Di Cataldo et al., 2023), dogs in Colombia (Santisteban-Arenas and Piedrahita, 2016), bush dogs (*Speothos venaticus*) in Argentina (Rinas et al., 2009), and dogs in Grenada (Chikweto et al., 2012). Most reports have been done with the observation of eggs in feces or post-mortem macroscopic observation of adult worms in lesions at necropsy, but molecular analyses have been implemented for species confirmation only is some of these reports (Gomez-Puerta et al., 2018). The importance of running both microscopic and molecular methods was demonstrated with the description of *Spirocerca vulpis*, a sister species of *S. lupi* that infects red foxes in Europe (Gama et al., 2020; Rojas et al., 2020a). *S. vulpis* is macroscopically undistinguishable from *S. lupi* but shows 30 um teeth in the buccal capsule and molecular divergence of 0.19-0.25% in the 18S rDNA and 9.34% in the cytochrome oxidase subunit 1 (*cox*1) when compared to *S. lupi* (Rojas et al., 2018b). In this setting, cryptic diversity within species can also be detected by using morphometric comparisons and mitochondrial and ribosomal markers (Chaves-Gonzalez et al., 2022). Importantly, *S. vulpis* has not been reported in the Americas, despite the introduction and range expansion of red foxes to North America in modern times (Hoffman and Sillero-Zubiri, 2021) and its finding in Portugal (Gama et al., 2020), Switzerland (Rojas et al., 2020a) and France (Medkour et al., 2020).

Molecular variation among *S. lupi* isolates has been observed in the internal transcribed spacer 1 (ITS1) loci and the *cox*1 gene, which has been useful to trace the species variability (Rojas et al., 2018a). Furthermore, *cox*1 sequences are easier to work with *S. lupi* than the highly variable ITS1, due to the great intraindividual variation observed in the ITS1 with up to six different copies of the loci in a single specimen (Rojas et al., 2018a). Accordingly, a phylogenetic study using the ITS1, *cox*1 and 18S rDNA loci, concluded that there is a high genetic variation among *S. lupi* specimens circulating in Africa, Asia, and Europe (Rojas et al., 2018a). The findings in the three loci led to the identification of two genotypes of *S. lupi*: the first genotype included specimens from Australia, India, Israel, and South Africa, and the second genotype with specimens from Hungary and Italy (Rojas et al., 2018a). Accordingly, a 0.14%, 8.06% and 6.48% variation in the 18S rDNA, ITS1 and *cox*1, respectively, were found between genotypes. However, the phylogenetic relationships of *S. lupi* in the Americas is not yet elucidated, and *cox*1 sequences from Andean foxes from Peru and Chile (Gomez-Puerta et al., 2018; Di Cataldo et al., 2023) confirmed the high genetic variability observed in geographical locations in the Americas. The present study analyzed *cox*1 and 18S rDNA gene fragments of specimens from Costa Rica, Mexico, and the United States and compared them with the specimens from the Old World.

## 2 Materials and methods

### 2.1 Specimen collection

A total of eight adult specimens of *S. lupi*, six from Costa Rica, three from Mexico and three from the US were obtained from domestic dogs. Two of these nematodes were found in subcutaneous tissues in a dog from Costa Rica (Porras-Silesky et al., 2022), and the other specimens were obtained from esophageal nodules after a *post-mortem* dissection or collection during endoscopic examination. Ethics Committee evaluation was not required since specimens were collected during routine animal evaluation without any intervention to patients.

### 2.2 DNA extraction

DNA was extracted using the DNeasy Blood and Tissue kit (Qiagen, Hilden, Germany), following the manufacturer’s instructions. Samples from Mexico and the US were incubated with 20 µL of proteinase K and 400 µL of ATL buffer at 56°C for 16 hours. DNA concentration and purity was measured in a NanoDrop (Thermo Scientific, US).

### 2.3 Amplification of the 18S rDNA and *cox*1 loci

Ribosomal 18S rDNA and mitochondrial *cox*1 genes were amplified in conventional PCRs. Analysis of the ITS1 loci was not included herein since genotyping with 18S rDNA and *cox*1 render the same genotyping results. In addition, *Spirocerca* spp. ITS1 have high intraindividual variability, leading to additional steps of cloning and sequencing (Rojas et al., 2018a).

To study the *cox*1 gene, two overlapping fragments were amplified. First, a 600 bp fragment, referred to as fragment A was amplified, using primers NTF (5’-TGATTGGTGGTTTTGGTAA-3’) and NTR (5’-ATAAGTACGAGTATCAATATC-3’) (Casiraghi et al., 2001) at 200 nM. Then, a 350 bp fragment, referred to as fragment B, was amplified using the JB3 (5’-TTTTTTGGGCATCCTGAGGTTTAT-3’) and JB4.5 (5’-TAAAGAAAGAACATAATGAAAATG-3’) (Bowles et al., 1992) primers at 400 nM. A fragment of approximately 850 bp of the 18S rDNA gene was amplified using the Nem18S rDNA-F (5’-CGCGAATRGCTCATTACAACAGC-3’) and Nem18S rDNA-R (5’-GGGCGGTATCTGATCGCC-3’) (Floyd et al., 2005) primers at 200 nM. All reactions were run at a final volume of 25 µL, 3 µL of DNA template, a positive control with *S. lupi* from Israel, a negative control of *Ehrlichia canis*, and a non-template control with ultra-purified water.

The obtained amplicons were examined on 1.5% agarose gels stained with SYBR-Safe at 90 V for 30 minutes. All DNA amplicons were purified with Exo-SAP (Thermo Scientific, US) and sequenced in both directions according to the Sanger method, using the ABI 3730xl System in Macrogen Inc., Korea.

### 2.4 Phylogenetic and species delimitation analyses

All sequences were inspected and trimmed using the MEGA 7.0 software (https://www.megasoftware.net/) (Kumar et al., 2016). The obtained 18S rDNA and *cox*1 sequences were aligned together with other *Spirocerca* spp. sequences, retrieved from the Genbank database using the MUSCLE algorithm (**Supplementary Tables 1**, **2**). Sequences corresponded to samples from Bosnia and Herzegovina, China, Hungary, India, Israel, Peru, South Africa, and Spain. Moreover, *Dirofilaria immitis* was used as an outgroup for both genes.

Data on 18S rDNA and *cox*1 genes were analyzed with Bayesian inference (BI) phylogenetic algorithm. First, the best nucleotide substitution models were calculated using the BI criteria of JModelTest 2.1.10 (Guindon and Gascuel, 2003; Darriba et al., 2012). The three-parameter model 2 with gamma distribution (TPM2+G) (Michalek and Ventura, 2019) was chosen for the 18S rDNA sequences and Tamura-Nei with gamma distribution (TrN+G) (Tamura and Nei, 1993) for the *cox*1. Phylogenetic trees were generated using the package Bayesian Evolutionary Analysis by Sampling Trees (BEAST v1.10.4) (Bouckaert et al., 2019), and an.xml input file was created with Bayesian Evolutionary Analysis Utility (BEAUti v1.10.4) with 10^7^ Markov chain Monte Carlo (MCMC) generations and a burn-in length of 10^3^. Tree priors were verified with Tracer v1.7.2, and trees were summarized using the TreeAnnotator v1.10.4. software and subsequently visualized with the FigTree 1.4.3 program. Moreover, maximum likelihood trees were traced using MEGA 10.0 to compare the topology to the BI tree. Additionally, a Templeton-Crandall-Sing (TCS) haplotype network was constructed to determine the phylogenetic relationships of the 18S rDNA and *cox*1 genes, with a 95% connection limit using the PopArt software (Leigh and Bryant, 2015).

### 2.5 Nucleotide distance and genetic structure analysis

Fragment B *cox*1 sequences were amplified in most (10 out of 14) *S. lupi* specimens, hence, were the most represented among geographical locations. A pairwise genetic nucleotide distance heatmap was constructed with fragment B *cox*1 sequences using the Sequence Demarcation Tool version 1.2 (http://web.cbio.uct.ac.za/~brejnev/) (Muhire et al., 2014). Additionally, a principal coordinate analysis (PCoA) was created using GenAIEx 6.503 to determine Nei´s genetic distance among fragment B *cox*1 sequences from different locations (Smouse et al., 2017). Pairwise nucleotide distances by using the p-distance method and bootstraps as the variance estimation method were calculated in MEGA 10.0.

To infer genetic structure, fragment B *cox*1 sequences from 28 *S. lupi* and five *S. vulpis* worms were analyzed using the Structure 2.3.4 software. Genetic population structure assigns individuals of the same species to a certain population or ‘*k* cluster’ based on genetic markers (Pritchard et al., 2000). The analysis was run with a burn-in length period of 5000 and several MCMC replicas after a burn-in of 10000 based on the MCMC algorithm. Moreover, ten simulations were performed for each *k* value (set from 1 to 10). (https://web.stanford.edu/group/pritchardlab/structure.html) (Hubisz et al., 2009). The optimum *k* value was determined using Structure harvester software (https://taylor0.biology.ucla.edu/structureHarvester/) (Earl and Vonholdt, 2012), which generated the in-files to use in the CLUMPP version 1.1.2 software. The CLUMPP software (https://rosenberglab.stanford.edu/clumpp.html) was used as a supporting software to identify the most frequent clustering patterns (Jakobsson and Rosenberg, 2007). Bar graphs of the clusters generated in CLUMPP for the optimum *k* value were plotted in Microsoft Excel.

### 2.6 Phylogeographic analysis

Phylogeographic analysis was done in all fragment B *cox1* sequences as also applied in other studies with eukaryotes (Tremetsberger et al., 2015; Vasquez-Aguilar et al., 2021; Andersen et al., 2022). A discrete phylogenetic diffusion analysis with the BEAST v1.10.4 software (Bouckaert et al., 2019) was applied. Briefly, BEAUti v1.10.4 was used to configure the parameters, including an asymmetric diffusion model for species and location (latitude and longitude) as traits. A Bayes factor test was implemented to identify the rates of the most parsimonious description of the phylogeographic diffusion process within categories of traits by considering a Bayesian stochastic search variable selection (BSSVS) analysis. The MCMC was run with a chain length of 2 x 10^8^ steps with a sub-sampling every 10 000 generations. The substitution model was set as a Hasegawa-Kishino-Yano (HKY) approach using 4 gamma rate categories. The approach included a constant size tree prior, and a strict molecular clock. Tracer v1.7.2 was used to monitor the convergence of the model with an Effective Sample Size ESS >200 for all parameters. Bayes factors were estimated using spreaD3_v0.9.7.1, in which values >10 were considered as strong evidence for diffusion events, as suggested before (Bouckaert et al., 2019). The resulting tree was annotated using the TreeAnnotator v1.10.4 software with a subsequent analysis in spreaD3_v0.9.7.1 to generate the phylogeographic map.

## 3 Results

### 3.1 Analysis of the 18S rDNA gene

**Figure 1:**
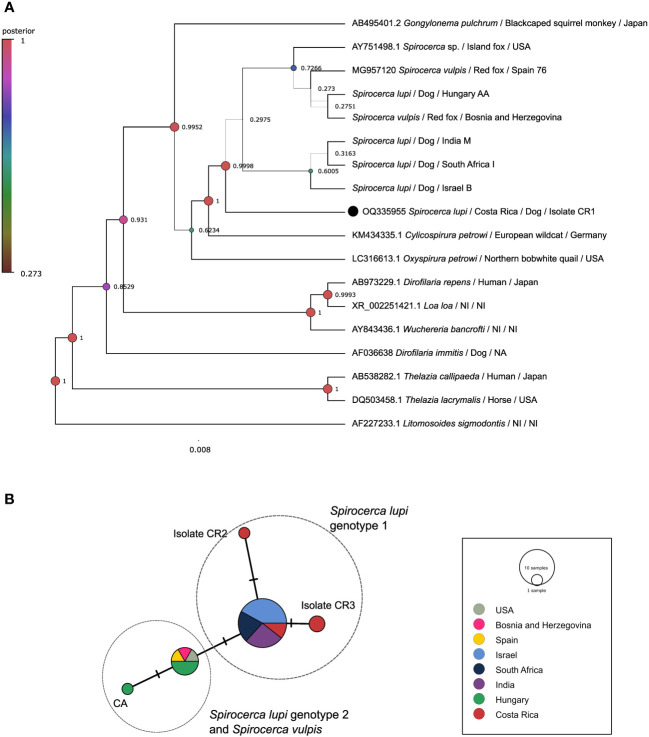
Analysis of the 18S rDNA gene. **(A)** Bayesian inference phylogenetic tree using a Three-parameter model 2 with gamma distribution for *Spirocerca* spp. and other species of the family Spiruridae. Posterior probability values are shown above the branches. **(B)** Templeton-Crandall-Sing (TCS) haplotype network, different colors correspond to the different sampling localities, and the size corresponds to the number of sequences that share the same haplotype. Mutations between sequences are shown as small lines in the branches. CR, Costa Rican specimens.

A 908 bp portion of the 18S rDNA gene in five *S. lupi* from Costa Rica was successfully amplified (**Supplementary Table 1**). In contrast, sequences of the specimens from Mexico and the USA could not be obtained possibly to low DNA concentration or fragmentation. Due to the low variability in this employed fragment, *S. lupi and S. vulpis* cannot be resolved. The BI tree clustered the Costa Rican specimens together with other *Spirocerca* spp. and closer to worms from Israel, South Africa, and India with 0.998 posterior probabilities (**Figure 1A**). The ML tree mirrored the topology of the BI analysis and sequences from Costa Rica were clustered with other *Spirocerca* spp (**Supplementary Figure 1A**).

The TCS haplotype network demonstrated five different *Spirocerca* haplotypes (**Figure 1B**). One haplotype is shared among sequences of *S. lupi* specimens from Costa Rica, Israel, India, and South Africa and another one is shared between *S. lupi* from Hungary and *S. vulpis* from Spain, Bosnia and Herzegovina, and USA sequences. Additionally, two independent haplotypes were represented by two sequences from Costa Rican specimens.

### 3.2 Analysis of the *cox*1 gene

**Figure 2:**
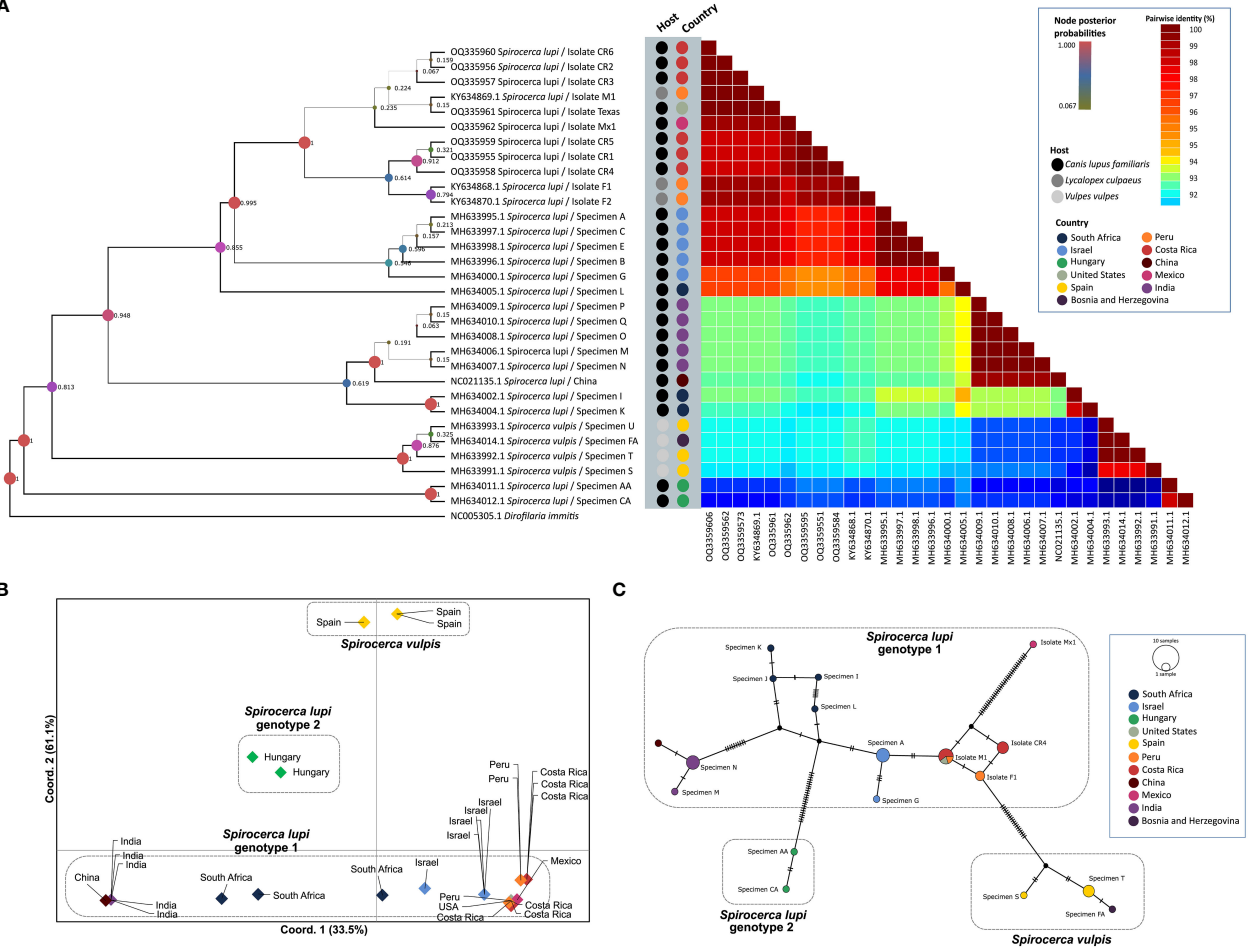
Analysis of *S. lupi cox*1 gene. **(A)** Bayesian inference phylogenetic tree based on the Tamura-Nei with gamma distribution model, inferred from fragment B of the *cox*1 gene. A color-coded heatmap of pairwise genetic distances of the *cox*1 fragment B sequences. The black, gray, and light gray dots refer to the host species of each sequence.(Pence and Stone) **(B)**. Principal coordinates analysis for the *cox*1 gene. **(C)** Templeton-Crandall-Sing (TCS) haplotype network for the *cox*1 gene, different colors correspond to the different sampling localities, and the size corresponds to the number of sequences that share the same haplotype. nucleotide substitutions are shown as small lines in the branches and hypothetical sequences are shown as black dots connecting the haplotypes.

Fragment B of the *cox*1 gene was amplified in 10 out of 14 *S. lupi* specimens from Costa Rica, Mexico, and the USA, whereas fragment A was amplified only for worms from Costa Rica. Therefore, this fragment was used for subsequent analyses. Two polymorphic sites among American sequences were observed in positions 115 and 154. When compared to Israeli sequences in the same fragment, five additional polymorphic sites were obtained, namely in positions 4, 49, 82, 157 and 196. Sequences from the American specimens (Costa Rica, Peru, Mexico, and USA) clustered in genotype 1, with a posterior probability of 0.995 in the BI phylogenetic tree (**Figure 2A**), very close to Israeli specimens. In addition, the same clustering of sequences was obtained in the ML tree (**Supplementary Figure 1B**), with *S. lupi* sequences from the Americas in the same group of Israel with 70 bootstraps. Moreover, the nucleotide distance heatmap showed a similarity from 97.74% to 99.35% (mean= 98.90%, standard deviation (SD)=0.47) between the specimens from the Americas and Israel. A lower pairwise identity was observed between specimens from the Americas and South Africa and India ranging from 94.52% to 98.38% similarity (mean=96.19%, SD=1.44). Nucleotide distance between *S. lupi* specimens from the Americas and *S. vulpis* showed 94.78% similarity (SD=0.22, range from 94.19 to 95.161). Meanwhile, nucleotide distance between *S. lupi* from the Americas and Hungary showed the lowest pairwise identity of all, with a range from 92.85% to 93.55% (mean=93.01%, SD=0.32). Pairwise nucleotide p-distance results are shown in **Supplementary File 1**.

The nucleotide distance PCoA confirmed previous findings by clustering sequences according to geographical locations (**Figure 2B**). Accordingly, a greater divergence was obtained between *S. vulpis* from Spain and *S. lupi* from Hungary compared to the divergence of *S. lupi* from Hungary to the other *S. lupi* genotype 1. Additionally, a close sequence association was observed between Israeli and American sequences. The greatest separation was observed in axis Y which separated genotypes 1 and 2 of *S. lupi* from *S. vulpis* sequences and explained 61.1% of the variance. Furthermore, axis X explained 33.5% of the observed variance and divided *S. vulpis* from both *S. lupi* genotypes. Sequences of specimens from China and India were clustered apart from the sequences obtained from Israeli and American specimens. Interestingly, sequences of South African nematodes were located between these two groups.

The TCS network showed 16 haplotypes and demonstrated a close association between the specimens from the Americas, as well as a shared haplotype of the Costa Rican, US, and Peruvian specimens (**Figure 2C**). Moreover, the specimen from Hungary, which was grouped in genotype 2 (Rojas et al., 2018a), presented a clear separation from the specimens that belonged to genotype 1 with 18 mutational steps. And, as expected, *S. vulpis* from Spain and Bosnia and Herzegovina created another cluster separated from *S. lupi* specimens.

**Figure 3:**
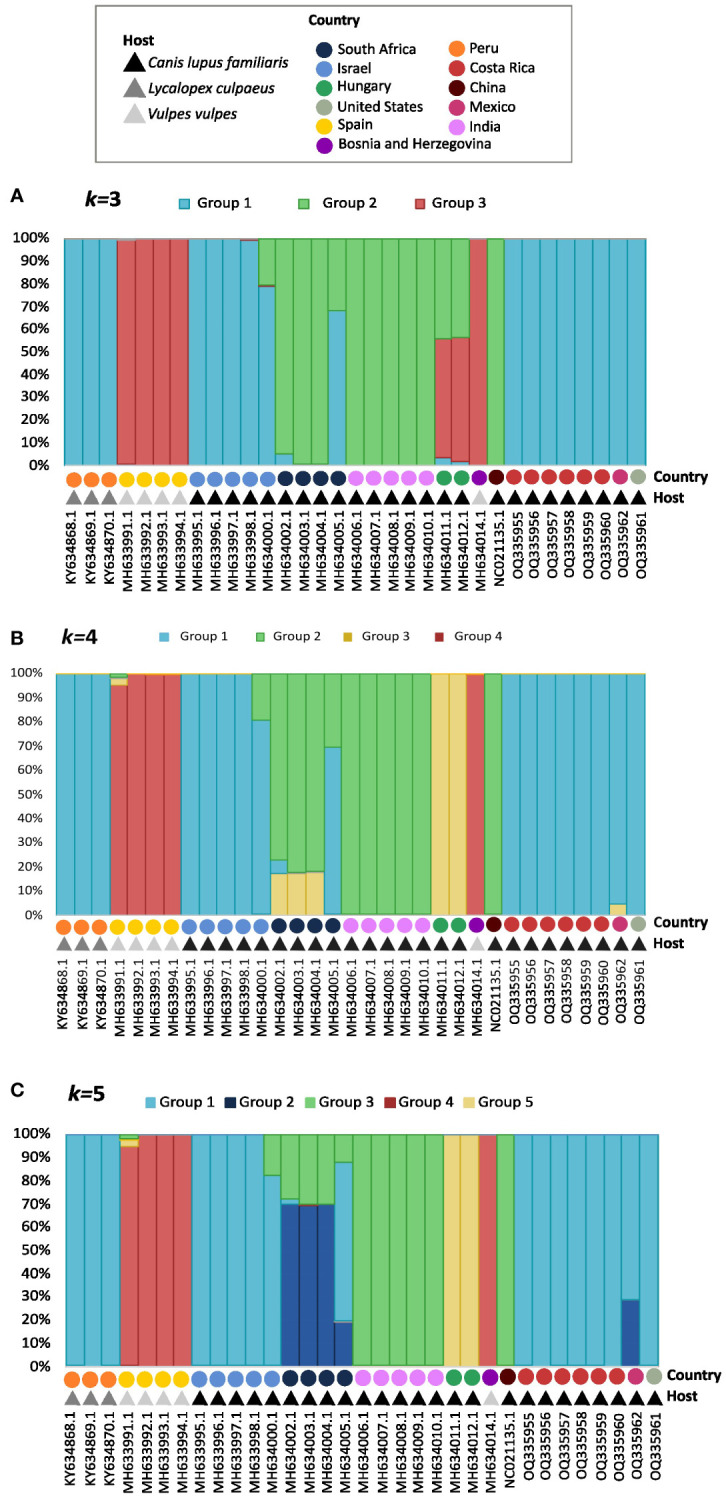
Graphs for the k=3 **(A)**, k=4 **(B)**, and k=5 **(C)** population structure results. Each bar represents an individual, which is identified by accession number, country, and host in the ‘x’ axis. The legend shows a different color for the hosts and geographic distributions for each of the 33 sequences of the cox1 gene. The different colors in the bars represent their probability of being a part of each group, which is shown in the `y` axis.

Population structure analysis defined *k*=3 as the optimum *k* value, so the populations were divided into 3 different groups or genetic clusters (**Figure 3A**). Group 1 contained all *S. lupi* sequences from the Americas, Israel, and partially one of the sequences from South Africa. In addition, group 2 clustered the sequences from India, China, and the other South African sequences, whereas group 3 contained sequences of *S. vulpis* from Spain. Interestingly, the two sequences from Hungary were placed in both group 2 and 3, approximately a 55% of the sequences belong to group 3, and 43% belong to group 2, the other 2% was placed in group 1. Likewise, graphs for the *k*=4 (**Figure 3B**) and *k*=5 (**Figure 3C**) were plotted, and the results were very similar to *k=3* but including additional subdivisions.

Four groups of sequences were estimated when using *k=4*: i) group 1 with sequences mainly from Israel, the Americas and one from South Africa, ii) group 2 with sequences mainly from South Africa, India and China, iii) group 3 formed by sequences from Hungary and iv) group 4 with *S. vulpis* sequences. The sequences from the Americas were not distinguishable from the Israeli, except for the Mexican sequence which shared a small percentage with group 3 from Hungary. Furthermore, all sequences from South Africa clustered in group 2, but in different percentages; however, one of these sequences shares only 30% with group 2, and 70% with other sequences in group 1.

When using *k=5 the* following groups were obtained (**Figure 3C**): i) group 1 with sequences from Israel and the Americas, ii) group 2 with a portion of South African and the Mexican sequence, iii) group 3 with sequences from India, China and portions of South Africa, iv) Group 4 with *S. vulpis and* v) group 5 with sequences from Hungary sequences.

### 3.3 Phylogeographic analysis

**Figure 4:**
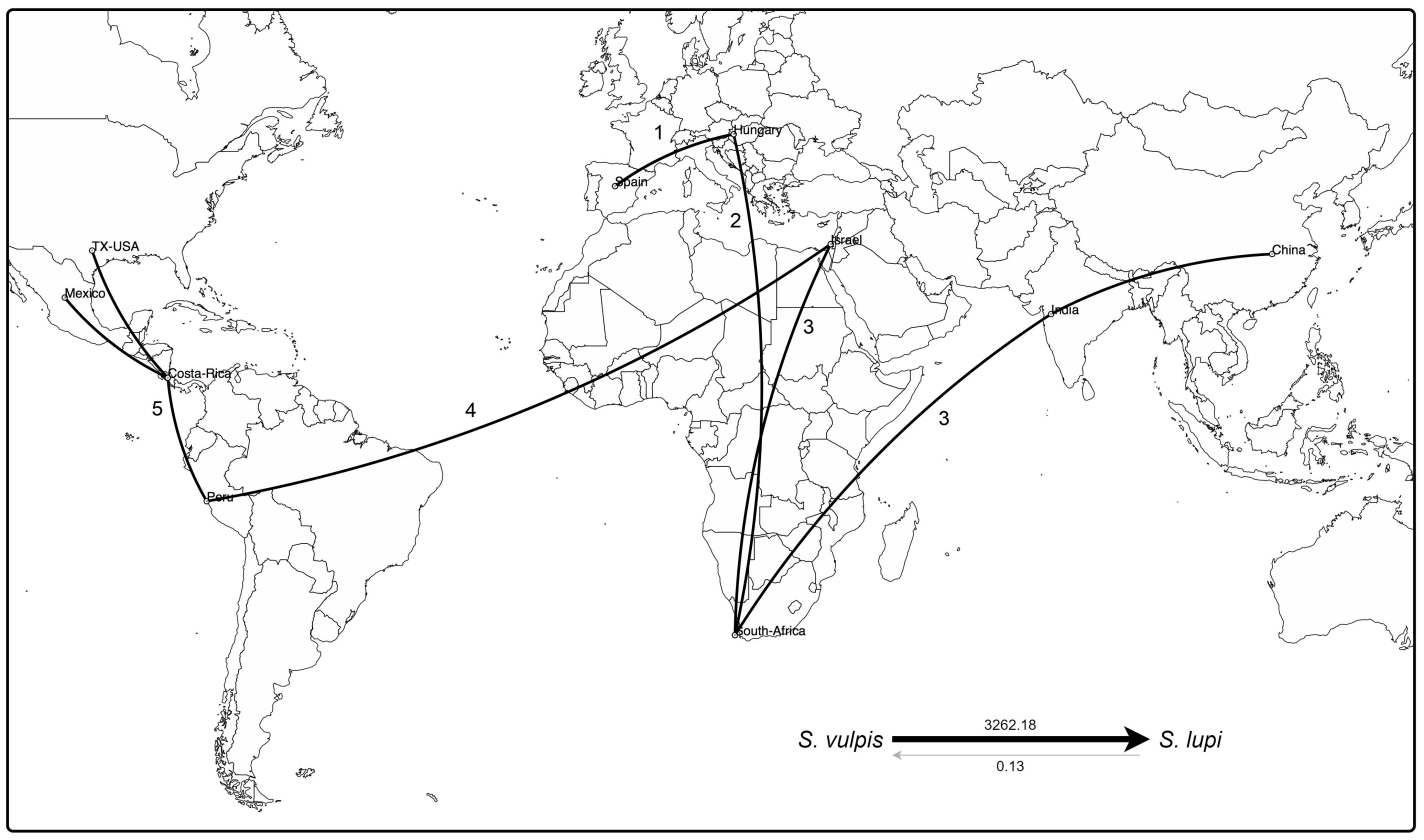
Phylogeographic analysis of *cox*1 sequences of *Spirocerca* spp. Distribution of the worm is suggested to have started from Europe to Africa and Asia, including the differentiation of the parental *S. vulpis* into *S. lupi*, as supported by the Bayes factors (right-bottom). An eminent arrival from Western Asia to the Americas is predicted, with a new genomic profile making Israel a hub element in the spread of *Spirocerca* spp. Numbers indicate spreading among geographic locations over time.

Based on the available sequences of *cox*1 gene (fragment B) and species and locations as traits, the discrete phylogenetic diffusion analysis suggests that *S. vulpis* sequences from Spain function as a starting point in the natural history of the distribution of *Spirocerca* spp. (**Figure 4**). This includes the differentiation of the ancestral *S. vulpis* into *S. lupi*, as supported by the Bayes factor value of 3262.18, in contrast to the opposite transition rate among species with a value <1 (**Figure 4**, right-bottom).

A first expansion seems to have occurred from Europe to Africa and subsequently to Asia in two different ways. Regarding the Chinese and Indian, sequences, these are highly related to sequences from Europe and South Africa, suggesting a first genetic group. In an independent way, a second group was predicted to have originated after the arrival of *Spirocerca lupi* to Israel leading to their spread to the Americas. In this second genetic cluster, the parasite probably arrived first to South America and then expanded northward into Central and North America. These results are in line with the predictions based on the TCS network, the phylogeny, and the nucleotide distance PcoA.

## 4 Discussion

The evolutionary history of the *Spirocerca* spp. has been speculated since the recent description of the *S. vulpis and* the characterization of different *S. lupi* genotypes. Phylogenetic analysis of *S. lupi* in the Americas has been limited, but our results shed light on the speciation process of this parasite. The data obtained in this study revealed that specimens from Costa Rica, Mexico, the United States, and Peru were closely related among each other, and to those from Israel according to *cox*1 analysis. Interestingly, our data at the phylogenetic and phylogeographic levels shows that *S. vulpis* from Europe was the common ancestor species from which *S. lupi* genotypes 1 and 2 derived and geographically expanded across Asia and Africa. According to the available data, it was shown that *S. lupi* from the Americas might have originated from Western Asia later in time. In fact, canids in the American continent, may have migrated across the Panama land bridge from North to South America (Chavez et al., 2022). Extant species in South America are monophyletic and have diversified throughout the years after being exposed to different environments, but the similarity in their diets and behaviors may have aided in the spread of *S. lupi* all over the continent (Chavez et al., 2022). Ribosomal 18S rDNA sequence analysis showed great similarities between *S. lupi* specimens from India, Israel, South Africa, and Costa Rica, as shown in the phylogenetic and haplotype network analyses. The 18S rDNA gene is a highly conserved and functional region, therefore, it evolves slower and is useful for phylogenetic reconstruction in higher taxonomic levels, but does not always discriminate among specimens from the same species (Kobayashi, 2011; Chan et al., 2021). Therefore, the 18S rDNA Bayesian phylogenetic tree showed that the *Spirocerca* spp. belong to a group that is separated from other members of the family Spiruridae as previously observed (Rojas et al., 2018b). This group includes two *S. vulpis* sequences and several *S. lupi* sequences, including the ones from Costa Rica. Since two *S. lupi* genotypes have been determined, mitochondrial genes were used in this study to attain a larger intraspecies resolution than that obtained for the 18S rDNA gene.

Mitochondrial DNA markers, such as the *cox*1, are suitable for species identification and establishing relationships between members of a population since they evolve fast in nematodes and, therefore, generate a higher degree of genetic variability than other molecular markers (Blouin, 2002; Chan et al., 2021). As pointed out before, molecular yardsticks for classifying cryptic diversity or separating species should adapt to each species, since each group has its own intrinsic variability (Chaves-Gonzalez et al., 2022). Despite the great variability in the ITS1 loci, *cox*1 sequences mirrors the clustering of the ITS1 (Rojas et al., 2018a), therefore, the latter is more practical to analyze. Bayesian inference phylogenetic tree, nucleotide distance PcoA, haplotype network and phylogeographic analyses for the *S. lupi cox*1 sequences confirmed the separation within the previously defined genotype 1 (Rojas et al., 2018a) into genotype 1A with sequences from specimens from the Americas and Israel and genotype 1B with sequences from South African, Indian and Chinese *S. lupi*. In addition, the results shown herein maintain the *S. lupi* genotype 2 with sequences of worms from Hungary (Rojas et al., 2018a). When comparing sequences from the Americas to the other sequences within genotype 1, the nucleotide distance heatmap showed a higher pairwise identity among the American *vs* Israeli specimens, than with Indian, Chinese, and South African specimens. Thus, verifying that the American sequences belong in the genotype 1A along with the Israeli sequences.

For the *cox*1 analysis of *S. lupi* it has been determined that the optimal number of *k* clusters is 3. Therefore, this analysis has been useful to categorize individuals into different populations based on objective features, such as genetic data, and not just on traits such as geographical location or hosts the specimens were found in (Pritchard et al., 2000). All specimens from the Americas, including the ones from Peru generated in another study, were categorized in Group 1 with the Israeli specimens in Genotype 1A. Group 2 contained worms from South Africa, India and China and Group 3 clustered with specimens of Bosnia and Herzegovina, Spanish, and Hungary. The results were very similar when using *k=*4 and *k=*5 clusters, however, one single individual can be a part of multiple clusters (Rosenberg et al., 2002), which was another interesting finding in this study with the South African and Mexican specimens. In *k=4*, the Mexican sequence showed a small percentage of divergence from Group 1 and a genetic similarity to Group 3, which mainly involves the Hungarian specimens (**Figure 3B**). In *k=5*, the Mexican sequences showed a genetic similarity to Group 2, which clusters the South African specimens (**Figure 3C**). Moreover, the PcoA and phylogeographic analyses confirmed the previous findings and clearly showed the separation of Genotype 1 into 1A and 1B. With these results, it can be inferred, once again, that the American specimens are more similar to the Israeli ones.

The Peruvian specimens, unlike the rest of the American specimens, were collected from Andean foxes, not domestic dogs, and the authors in this study found less than 5% nucleotide difference between the Peruvian and African, Asian, and European sequences (Gomez-Puerta et al., 2018). The results obtained there were fairly similar to ours, when comparing the short sequences of the *cox*1 (Gomez-Puerta et al., 2018). These sequences also showed a 98-99% similarity to the other sequences from the Americas. This high similarity might be explained by the fact that *S. lupi* infected a myriad of canid species, including Andean foxes due to their phylogenetic proximity, and lack of host specificity. Andean foxes might have become infected with *S. lupi* as a spillover effect from infected dogs or other unrecognized or extinct infected canid hosts in the area (Guzmán-Sandoval et al., 2007; Gomez-Puerta et al., 2018). However, there is still much we don’t know about *S. lupi* host-specificity in the Americas. For example, a study in South Africa found that black-backed jackals (*Canis mesomelas*) are less susceptible than dogs to the parasite (Bumby et al., 2017), but the role of Andean foxes and other canids in the distribution and cycle of *S. lupi* in the Americas is not well elucidated yet. Therefore, to further understand how *S. lupi* circulates in this region, more epidemiological and molecular studies are needed.

One of the most interesting questions that derive from this study is the geographic expansion route of *S. lupi* between the continents, especially if the parasite migrated from Israel to America as suggested by the phylogeographic approach. The migration from one continent to another is not unique to *S. lupi*. Small et al. (Small et al., 2019), studied the spread of the human filarioid *Wuchereria bancrofti* through the tropics by using whole genome DNA sequences, finding that this species probably arrived to Haiti (New World) from Africa along with people during the transatlantic slave trade. In addition, the authors proposed that the genetic diversity among these parasite populations could be due to the different environments, vectors and variations in the immune response of the different hosts (Small et al., 2019). Similarly, Laidoudi et al. (2022), also highlighted the importance of the epidemiological context when discussing genetic diversity among vector-borne helminths of canids, as well as the difference in the vectors and hosts involved (Laidoudi et al., 2022). The results obtained in the *W. bancrofti* analysis are highly robust due to the study of the whole set of genes of several isolates. On the other hand, herein we could analyze a single mitochondrial locus from more than 20 different specimens since the genome of *S. lupi* has not been sequenced yet. Therefore, conclusions drawn from the present work should be interpreted cautiously. Nevertheless, to further explain the migration of *S. lupi*, when it spread through the Americas and the genetic diversity established, more epidemiological studies with more comprehensive, geographic and host sampling, molecular characterization, and more robust molecular datasets is needed.

Phylogeographic tools employed herein have been designed for the analysis of the evolutionary processes of rapidly changing viruses and bacteria (Bielejec et al., 2016). Importantly, these methods have been used before in the analysis of other organisms like the protozoa *Hepatozoon* spp. (Vasquez-Aguilar et al., 2021) or Northern Birch mice (Andersen et al., 2022) using longer sequences such as mitochondrial DNA or a higher number of specimens. Therefore, a major limitation of this study relies in the analysis of a lower number of *S. lupi* sequences due to the difficulty in obtaining whole specimens from dog lesions. Moreover, similar phylogeographic algorithms designed specifically for helminths are scarce or do not exist. The presented results must be interpreted with caution since mutational rates and generational times vary with genome sizes and will vary in viruses and bacteria (Lynch, 2010), and in this case, in nematodes. The herein analyses highlight the use and limitations of bioinformatic applications and the adaptation of these tools to different biological groups, such as helminths.

## 5 Conclusions

*Spirocerca lupi* collected from three geographic areas of the Americas belongs to genotype 1 according to the analysis of a *cox*1 fragment *and* is closely related to specimens from Israel, as inferred using a combination of phylogenetic, haplotype and phylogeographic analyses. Additionally, it is suggested that the *Spirocerca* spp. evolutionary history began with *S. vulpis in* Eurasia, leading to its divergence into *S. lupi* which later spread to Africa, Asia, and South America, along with canid hosts. This study analyzed a single locus of 32 *S. lupi and S. vulpis* specimens from 11 different geographical regions of the world, and thus, these results should be interpreted with caution since phylogeographic algorithms are designed for bacterial or viral genomes. This in turn, highlights the need for bioinformatic tools tailored to reflect the evolutionary history of helminths.

## Data availability statement

The original contributions presented in the study are included in the article/**Supplementary files**, further inquiries can be directed to the corresponding author.

## Ethics statement

The manuscript presents research on animals that do not require ethical approval for their study.

## Author contributions

PA-S, JR-Q and AR conceived the study. VM-H, GV and RR-V provided *S. lupi* specimens. PA-S, JR-Q, JM-M, GB, and AR analyzed the data and generated the figures. PA-S and AR wrote the first draft of the manuscript. All authors critically reviewed and edited the manuscript and approved its final version.

## Supplementary material

Supplementary Figure 1 Maximum likelihood trees of 18S rDNA **(A)** and *cox*1 **(B)** gene fragments based on the Tamura-Nei with gamma distribution model. Bootstrap values below 70 are now shown.

Supplementary File 1 Nucleotide pairwise p-distance among *S. lupi and S. vulpis cox*1 sequences included in the study.
