# Supplementary figures and images for: Elucidating Spirocerca lupi spread in the Americas by using phylogenetic and phylogeographic analyses
Source: Front Parasitol. 2023 Sep 27;2:1249593. doi: 10.3389/fpara.2023.1249593 (PMC11731684; doi:10.3389/fpara.2023.1249593)

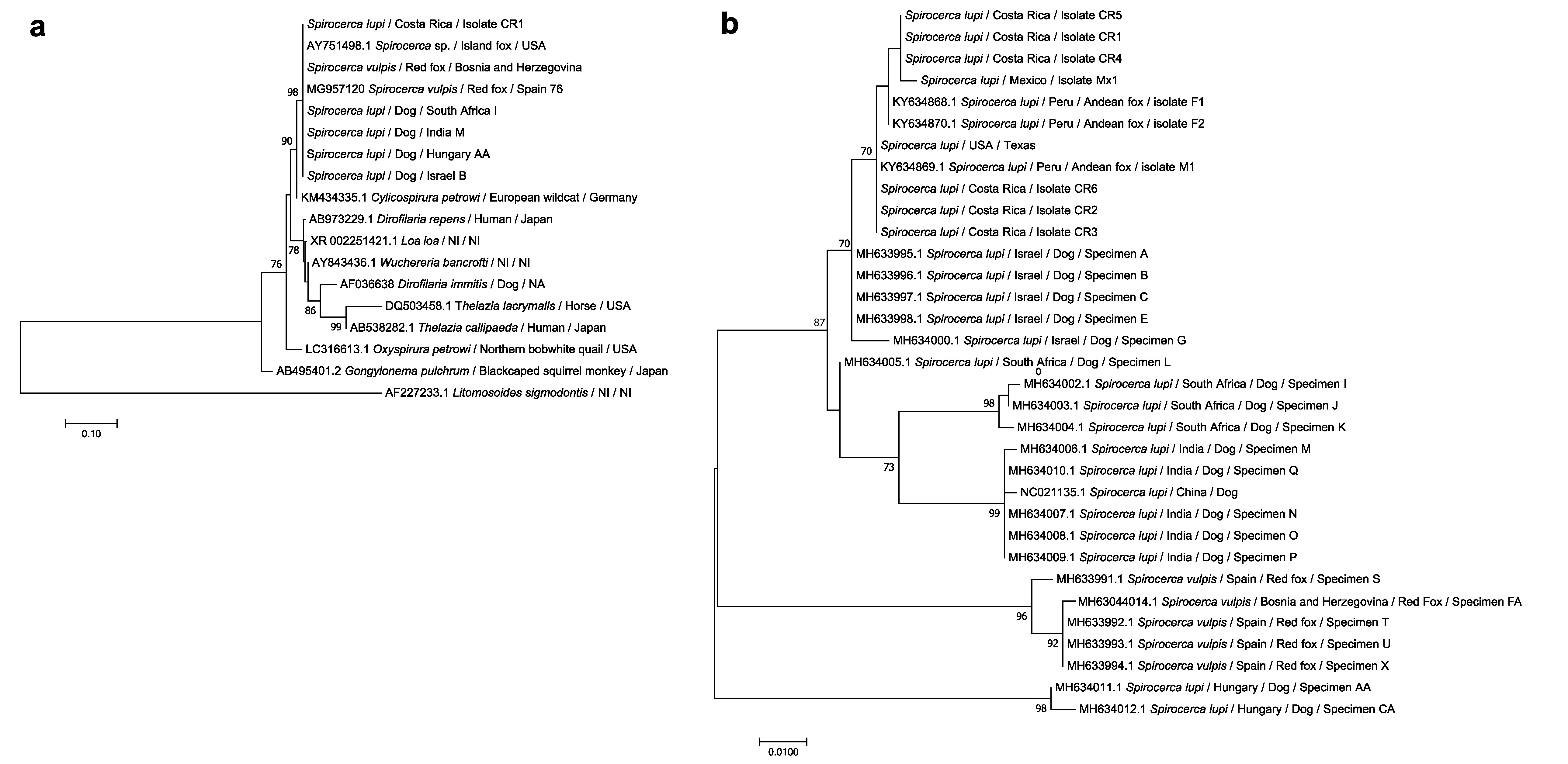

Supplement: Supplementary Figure 1 — Maximum likelihood trees of 18S rDNA (A) and cox1 (B) gene fragments based on the Tamura-Nei with gamma distribution model. Bootstrap values below 70 are now shown. [file Image_1.jpeg]
