# Supplementary material for: Elucidating Spirocerca lupi spread in the Americas by using phylogenetic and phylogeographic analyses
Source: Front Parasitol. 2023 Sep 27;2:1249593. doi: 10.3389/fpara.2023.1249593 (PMC11731684; doi:10.3389/fpara.2023.1249593)
Supplement: Supplementary File 1 — Nucleotide pairwise p-distance among S. lupi and S. vulpis cox1 sequences included in the study. [file Table_1.docx]

Supplementary Table 1. Summary of *S. lupi* 18S sequences used in the analysis.

| Species | Country of origin | Host | Internal code | Accession number |
| --- | --- | --- | --- | --- |
| *Spirocerca* sp*.* | USA | Island fox | USA | AY751498.1 |
| *S. lupi* | Hungary | Dog | Hungary_AA | MH634011 |
|  | Hungary | Dog | Hungary_CA | MH634012 |
|  | Hungary | Dog | Hungary_DA | MH634013 |
|  | Israel | Dog | Specimen_B | MH633996.1 |
|  | Israel | Dog | Specimen_C | MH633997.1 |
|  | Israel | Dog | Specimen_E | MH633998.1 |
|  | Israel | Dog | Specimen_F | MH633999 |
|  | Israel | Dog | Specimen_G | MH634000.1 |
|  | Israel | Dog | Specimen_H | MH634001 |
|  | India | Dog | Specimen_M, | MH634006.1 |
|  | India | Dog | Specimen_N, | MH634007.1 |
|  | India | Dog | Specimen_O, | MH634008.1 |
|  | India | Dog | Specimen_P | MH634009.1 |
|  | India | Dog | Specimen Q | MH634010.1 |
|  | South Africa | Dog | Specimen_I, | MH634002.1 |
|  | South Africa | Dog | Specimen_J, | MH634003.1 |
|  | South Africa | Dog | Specimen_K | MH634004.1 |
|  | South Africa | Dog | Specimen_L | MH634005.1 |
|  | Costa Rica | Dog | Isolate_CR1 | OQ335955 |
|  | Costa Rica | Dog | Isolate_CR2 | OQ335956 |
|  | Costa Rica | Dog | Isolate_CR3 | OQ335957 |
|  | Costa Rica | Dog | Isolate_CR4 | OQ335958 |
|  | Costa Rica | Dog | Isolate_CR5 | OQ335959 |
| *S. vulpis* | Spain | Red fox | Spain_76 | MG957120 |
|  | Bosnia and Herzegovina | Red fox | Bosnia_and_Herzegovina | MH634014 |
