# Supplementary material for: Elucidating Spirocerca lupi spread in the Americas by using phylogenetic and phylogeographic analyses
Source: Front Parasitol. 2023 Sep 27;2:1249593. doi: 10.3389/fpara.2023.1249593 (PMC11731684; doi:10.3389/fpara.2023.1249593)
Supplement: Supplementary file 3 [file Table_2.docx]

Supplementary Table 2. Summary of *S. lupi* *cox*1 sequences used in the analysis .

| Species | Country of origin | Host | Internal code | Accession number |
| --- | --- | --- | --- | --- |
| *S. lupi* | Mexico | Dog | Isolate_Mx | OQ335962 |
|  | Peru | Andean fox | Isolate_M1 | KY634869.1 |
|  | Peru | Andean fox | Isolate_F1 | KY634868.1 |
|  | Peru | Andean fox | Isolate_F2 | KY634870.1 |
|  | Costa Rica | Dog | Isolate_CR1 | OQ335955 |
|  | Costa Rica | Dog | Isolate_CR2 | OQ335956 |
|  | Costa Rica | Dog | Isolate_CR3 | OQ335957 |
|  | Costa Rica | Dog | Isolate_CR4 | OQ335958 |
|  | Costa Rica | Dog | Isolate_CR5 | OQ335959 |
|  | Costa Rica | Dog | Isolate_CR6 | OQ335960 |
|  | USA | Dog | Isolate_Tx | OQ335961 |
|  | Israel | Dog | Specimen_A | MH633995.1 |
|  | Israel | Dog | Specimen_B | MH633996.1 |
|  | Israel | Dog | Specimen_C | MH633997.1 |
|  | Israel | Dog | Specimen_E | MH633998.1 |
|  | Israel | Dog | Specimen_G | MH634000.1 |
|  | South Africa | Dog | Specimen_I | MH634002 |
|  | South Africa | Dog | Specimen_K | MH634004 |
|  | South Africa | Dog | Specimen_L | MH634005.1 |
|  | India | Dog | Specimen_M | MH634006.1 |
|  | India | Dog | Specimen_N | MH634007.1 |
|  | India | Dog | Specimen_O | MH634008.1 |
|  | India | Dog | Specimen_P | MH634009.1 |
|  | India | Dog | Specimen_Q | MH634010.1 |
|  | China | Dog | China | NC021135.1 |
|  | Hungary | Dog | Specimen_AA | MH634011.1 |
|  | Hungary | Dog | Specimen_CA | MH634012.1 |
| *S. vulpis* | Bosnia and Herzegovina | Red fox | Specimen_FA | MH634014.1 |
|  | Spain | Red fox | Specimen_S | MH633991. |
|  | Spain | Red fox | Specimen_T | MH633992.1 |
|  | Spain | Red fox | Specimen_U | MH633993.1 |
|  | Spain | Red fox | Specimen_X | MH633994.1 |
